# Supplementary material for: Repurposing the mitotic machinery to drive cellular elongation and chromatin reorganisation in Plasmodium falciparum gametocytes
Source: Nat Commun. 2022 Aug 27;13:5054. doi: 10.1038/s41467-022-32579-4 (PMC9419145; doi:10.1038/s41467-022-32579-4)
Supplement: Supplementary file 1 — Supplementary Information [file 41467_2022_32579_MOESM1_ESM.pdf]

SUPPLEMENTARY INFORMATION

Repurposing the mitotic machinery to drive cellular elongation and chromatin reorganisation in *Plasmodium falciparum* gametocytes

Jiahong Li[1#], Gerald J. Shami[1#], Ellie Cho[1,2], Boyin Liu[1], Eric Hanssen[1,3], Matthew W.A. Dixon[4,5*], Leann Tilley[1*]

[1]Department of Biochemistry and Pharmacology, Bio21 Molecular Science and Biotechnology Institute, The University of Melbourne, Parkville, VIC 3010, Australia; [2]Biological Optical Microscopy Platform, The University of Melbourne, Parkville, VIC 3010, Australia; [3]Ian Holmes Imaging Center, Bio21 Molecular Science and Biotechnology Institute, The University of Melbourne, Parkville, VIC 3010, Australia; [4]Department of Infectious Diseases, The Peter Doherty Institute, The University of Melbourne, Parkville, VIC 3010, Australia; [5]Walter and Eliza Hall Institute, Parkville, VIC, 3010, Australia.

This PDF file includes:
Supplementary Tables 1 to 4
Supplementary Figures 1 to 14
Supplementary References

Supplementary Tables

Supplementary Table 1. Mander's coefficients (M) for co-occurrence of kinetochore, centromere and Hoechst fluorescence signals in different stage gametocytes.

Stage	***Pf*NDC80/ *Pf*CENH3 (M)**	***Pf*CENH3/ *Pf*NDC80 (M)**	**Hoechst/ *Pf*CENH3 (M)**	***Pf*CENH3/ Hoechst (M)**	**Hoechst/ *Pf*NDC80 (M)**	***Pf*NDC80/ Hoechst (M)**
**I**	0.64 ± 0.23	0.40 ± 0.21	0.52 ± 0.26	0.49 ± 0.19	0.17 ± 0.11	0.30 ± 0.21
**II**	0.63± 0.27	0.45 ± 0.21	0.63 ± 0.27	0.57 ± 0.18	0.31 ± 0.17	0.41 ± 0.25
**III**	0.75 ± 0.19	0.57 ± 0.20	0.70 ± 0.23	0.54 ± 0.18	0.47 ± 0.20	0.52 ± 0.21
**IV**	0.83 ± 0.21	0.66 ± 0.24	0.19 ± 0.17	0.67 ± 0.21	0.10 ± 0.07	0.60 ± 0.28
**V**	0.88 ± 0.13	0.82 ± 0.11	0.13 ± 0.07	0.58 ± 0.26	0.12 ± 0.11	0.47 ± 0.28

The analysis relates to the data in Fig. 4a. Data represent mean ± SD; n = 20 cells for each stage. Source data is provided in the Source Data file.

Supplementary Table 2. Statistical analysis of differences in Mander's coefficient (M) for co-occurrence of kinetochore, centromere and Hoechst fluorescence signals in different stage gametocytes.

**Mander's coefficient (M)**	**I vs. IV Summary P value**	**I vs. V Summary P value**	**II vs. IV Summary P value**	**II vs. V Summary P value**	**III vs. IV Summary P value**	**III vs. V Summary P value**
*Pf*NDC80/*Pf*CENH3	* 0.0417	** 0.0033	* 0.0354	** 0.0027	NS 0.8016	NS 0.2964
*Pf*CENH3/*Pf*NDC80	*** 0.0004	**** < 0.0001	** 0.0077	**** < 0.0001	NS 0.5245	*** 0.0007
Hoechst/*Pf*CENH3	**** < 0.0001	**** < 0.0001	**** < 0.0001	**** < 0.0001	**** < 0.0001	**** < 0.0001
*Pf*CENH3/Hoechst	NS 0.0525	NS 0.6003	NS 0.5399	NS 0.9994	NS 0.2480	NS 0.9468
Hoechst/*Pf*NDC80	NS 0.4971	NS 0.6843	**** < 0.0001	*** 0.0002	**** < 0.0001	**** < 0.0001
*Pf*NDC80/Hoechst	** 0.0026	NS 0.1967	NS 0.1168	NS 0.9172	NS 0.8547	NS 0.9767

Differences in Mander's coefficients (Supplementary Table 1) were determined by unpaired One-way ANOVA Turkey's test. Source data is provided in the Source Data file.

Supplementary Table 3. Primers used for generation of reporter constructs (restriction sites in bold)

**Primer name**	**Sequence (5'-3')**
XhoI-*Pf*EB1-Fw	tttcttacatataa**CTCGAG**ATGACTGAATATAAAGACTTACAAACAGC
KpnI-*Pf*EB1-Re	tcttctcctttact**GGTACC**TGAACAGTAGGTGGCGTAGTC
XhoI-*Pf*Centrin-1-Fw	tttcttacatataa**CTCGAG**ATGAGCAGAAAAAATCAAACTATGAT
KpnI-*Pf*Centrin-1-Re	gcccttgctcaccat**GGTACC**AAATAAGTTGGTCTTTTTCATAATTCTCA
XhoI-*Pf*Centrin-4-Fw	tttcttacatataa**CTCGAG**ATGAACACAATGTTAATTAAGGATAACAT
KpnI-*Pf*Centrin-4-Re	gcccttgctcaccat**GGTACC**TGAATCTGAATCAACATCACTTAAT
XhoI-*Pf*NDC80-Fw	ttagtttccag**CTCGAG**ATGAATAATCATTCAGTGTATAATAAATTC
KpnI-*Pf*NDC80-Re	cttgctcaccat**GGTACC**ATCAACAACACTTTTTTGAAG
test-*Pf*NDC80-Fw	GAAATAAAGTTCTTTGTGAGG
NotI-5'FR-*Pf*NDC80-Fw	**GCGGCCGC**GCGAAACTATCTATCAAATTGAAC
XhoI-5'FR-*Pf*NDC80-Re	gaatgattattcat**CTCGAG**CTGGAAACTAAACGAAATTG
test-5'FR-*Pf*NDC80-Fw	GTATTGTCCTTCAACATATTTG
XhoI-GFP-Fw	gaatagaataaa**CTCGAGATG**AGTAAAGGAGAAGAACTTTTCAC
KpnI-GFP-Rev	gttctcaccat**GGTACC**TTTGTATAGTTCATCCATGCCATG
KpnI-*Pf*CENH3-Fw	ctatacaaa**GGTACC**ATGGTGAGAACAAAAAAGAATATACC
PacI-*Pf*CENH3-stop codon-Re	ccatatcc**TTAATTAA**TTAGTCTCTTCCGCGTATTC

Supplementary Table 4. Transgenic parasite reporters generated in this study.

Transfectant name	Structure labelled	Parental parasite lines
*Pf*EB1-GFP	Nuclear microtubules	pCRT-*Pf*EB1-GFP (WR)
NLS-FRB-mCherry (NLS-mCherry)[1]	Nuclear lumen	pNLS-mCherry (DSM1)
*Pf*EB1-GFP/NLS- mCherry	Nuclear microtubules, lumen	pCRT-*Pf*EB1-GFP (WR), pNLS-mCherry (BSD)
*Pf*centrin-4-mCherry	centriolar plaque/ MTOC	pCRT-*Pf*centrin-4-mCherry (BSD)
*Pf*centrin-1-mCherry	centriolar plaque/ MTOC	pCRT-*Pf*centrin-1-mCherry (BSD)
*Pf*NDC80-mCherry	Kinetochore	p*Pf*NDC80-*Pf*NDC80-mCherry (BSD)
GFP-*Pf*CENH3	Centromere	p*Pf*NDC80-GFP-*Pf*CENH3 (WR)
*Pf*centrin-1-mCherry/ *Pf*EB1-GFP	centriolar plaque/ MTOC, nuclear microtubules	pCRT-*Pf*centrin-1-mCherry (BSD), pCRT-*Pf*EB1-GFP (WR)
*Pf*centrin-4-mCherry/*Pf*EB1-GFP	centriolar plaque/ MTOC, nuclear microtubules	pCRT-*Pf*centrin-4-mCherry (BSD), pCRT-*Pf*EB1-GFP (WR)
*Pf*NDC80-mCherry/*Pf*EB1-GFP	Kinetochore, nuclear microtubules	p*Pf*NDC80-*Pf*NDC80-mCherry (BSD), pCRT-*Pf*EB1-GFP (WR)
GFP-*Pf*CENH3/*Pf*NDC80-mCherry	Centromere, kinetochore	p*Pf*NDC80-GFP-*Pf*CENH3 (WR), p*Pf*NDC80-*Pf*NDC80 -mCherry (BSD)
GFP-*Pf*CENH3/*Pf*centrin-4-mCherry	Centromere, centriolar plaque/ MTOC	p*Pf*NDC80-GFP-*Pf*CENH3 (WR), pCRT-*Pf*centrin-4-mCherry (BSD)

Supplementary Figures

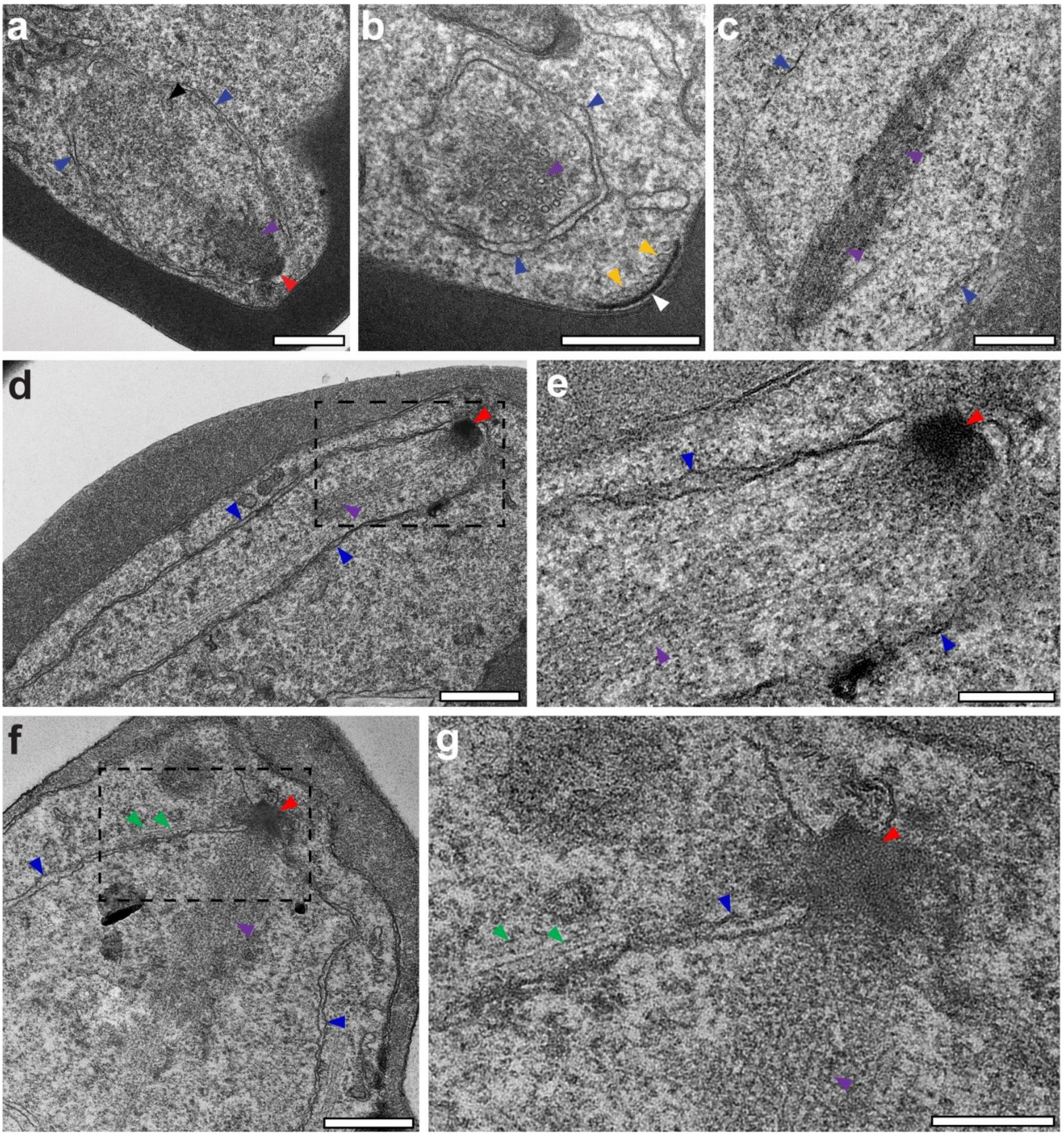

Supplementary Figure 1. Transmission electron microscopy survey of nuclear microtubule architecture in stage II/III gametocytes. Thin section electron micrographs illustrating different features in the gametocyte nucleus. Microtubule bundles (purple arrowheads) in transverse- **a**, **c-g** or cross- **b** sections are located within the nuclear membrane (dark blue arrowheads). The microtubules arise from an amorphous electron-dense MTOC (red arrowheads) that is embedded in the nuclear membranes. Chromatin material is observed associated with the microtubules (**a**, black arrowhead). Cytoplasmic microtubules emanate from the outer centriolar plaque (**f**, **g**, green arrowheads). The experiments were performed 3 times. Scale bars: 200 nm.

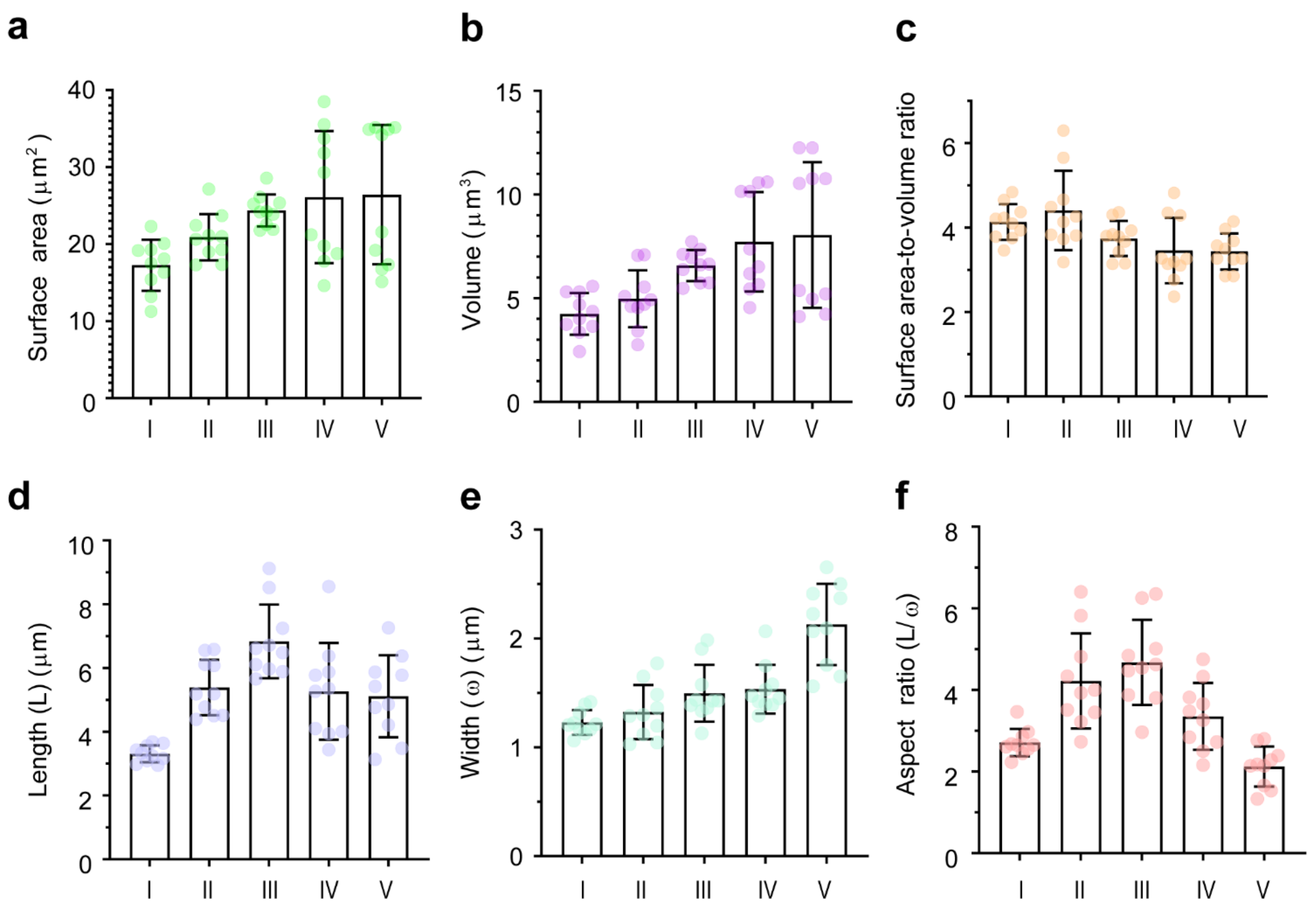

Supplementary Figure 2. Morphometric analysis of the nuclear compartments during gametocyte development. An array tomography survey of stage I-V gametocytes was performed and the surface area, volume, length and width of the nucleus in each of the stages measured. The aspect ratios of surface area to volume and length to width are also shown. The error bars are mean ± SD, n = 10 cells for each lifecycle stage. Pixel size, x, y, z: 4.2 x 4.2 x 120 nm. Source data is provided in the Source Data file.

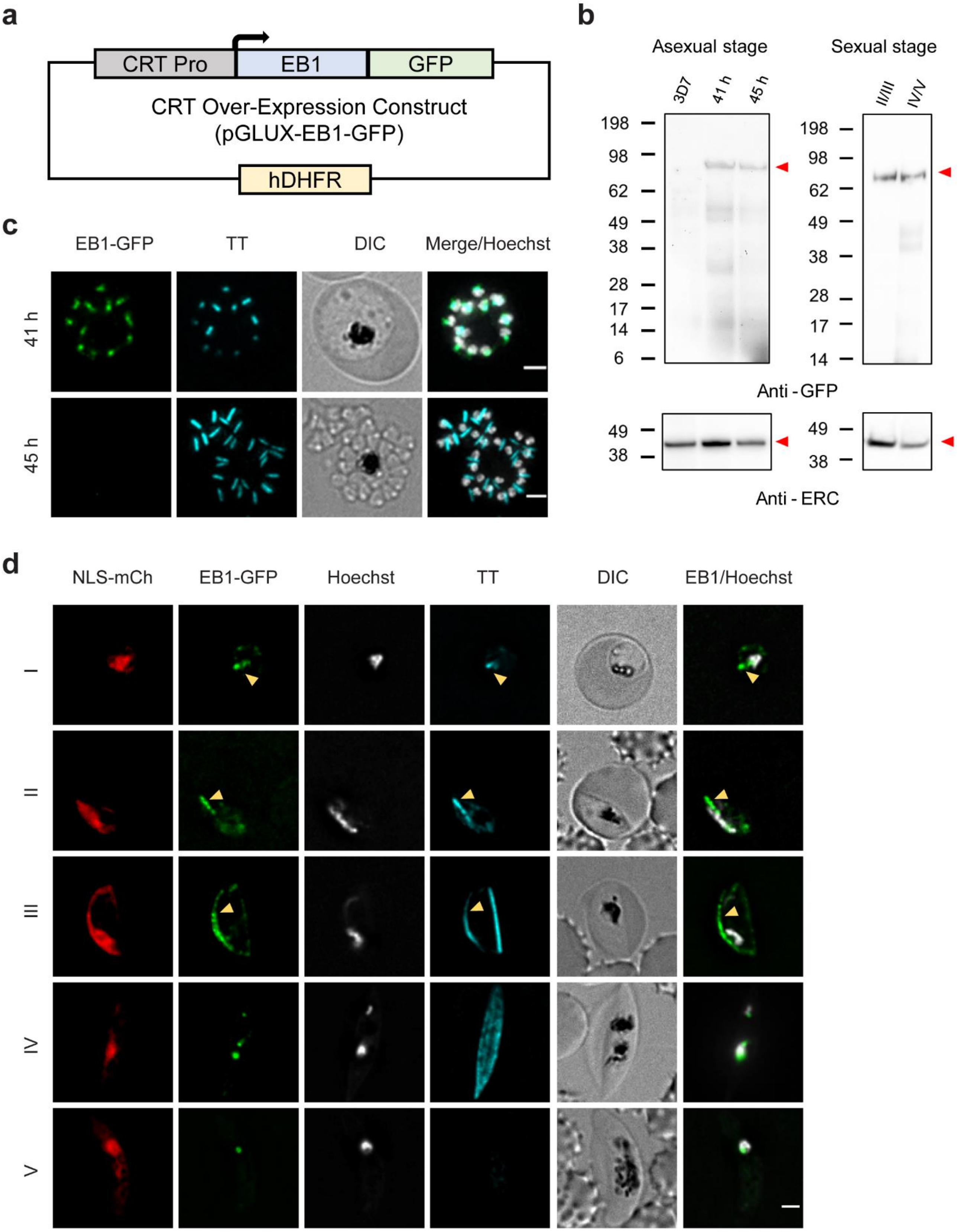

Supplementary Figure 3. End-binding protein-1 as a marker of nuclear but not sub-pellicular microtubules. a Illustration of the construct used to generate the *Pf*EB1-GFP transfectants. **b** Western blots of asexual schizonts and stage II, III, IV, V gametocytes showing *Pf*EB1-GFP at the expected molecular mass (red arrows). The parent cell line (3D7) was used as the control. *Pf*ERC was used as a

loading control for the Western blots. **c** Live cell fluorescence imaging of asexual blood stage schizont (41 h) showing short rods of *Pf*EB1-GFP (EB1-GFP, green), likely delineating the spindles in the dividing nuclei, as marked by Tubulin Tracker (TT, cyan) and Hoechst (grayscale). **d** Fluorescence microscopy images of live stage I to stage V gametocytes from the *Pf*EB1-GFP/NLS-mCherry co-transfectant parasite line. NLS-mCherry (NLS-mCh, red) delineates the nucleus. *Pf*EB1-GFP (EB1-GFP, green) marks the nuclear microtubules (yellow arrows). Tubulin Tracker (TT, cyan) labels both the nuclear and subpellicular microtubule populations. Hoechst (grayscale) labels the chromatin. Differential interference contrast (DIC) images are shown. Scale bars: 2 μm. The Western blot analysis was performed 3 times. Each of the asexual and sexual stage live cell imaging experiments were performed 3 times.

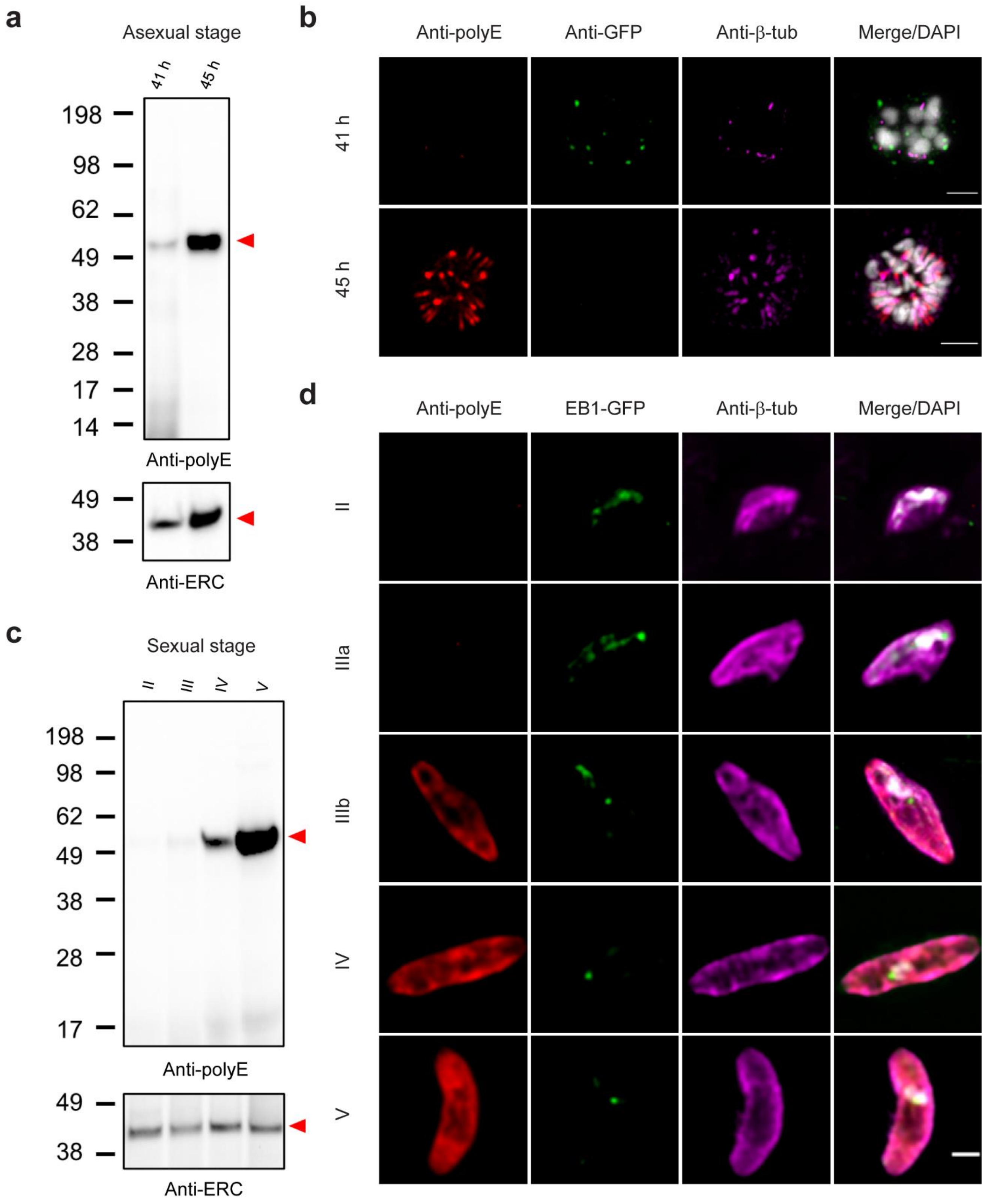

Supplementary Figure 4. Sub-pellicular microtubules are stabilised by post-translational modification with polyglutamate. Western analysis using an antibody recognising polyglutamate (polyE) showing a band at the expected molecular mass for β-tubulin. A strong signal is observed in asexual mature schizonts (**a**), and late stage gametocytes (**c**). **b**, **d** Immunofluorescence microscopy images of *P. falciparum* transfectant asexual schizonts (**b**) and stage II-V gametocytes (**d**) expressing

*Pf*EB1-GFP, which marks the nuclear microtubules. The *Pf*EB1-GFP signal was amplified using anti-GFP (green) (**b**) or measured directly as the remnant *Pf*EB1-GFP (EB1-GFP, green) signal (**d**). The *Pf*EB1-GFP signal reveals that the nuclear microtubules are depolymerised in mature schizonts and in stage IIIb/IV/V gametocytes. Anti-polyE (red) labelling shows that the subpellicular microtubules are modified with polyE in mature schizonts and in stage IIIb/IV/V gametocytes. The microtubules were labelled with anti-β-tub (magenta) and the chromatin was labelled with DAPI (grayscale). Panel **d** represents additional examples of the data presented in Fig. 2d. Panel **b** images were generated using a Zeiss Elyra LSM880 confocal microscope. Panel **d** images were acquired using a DV Elite deconvolution microscope. Scale bars: 2 μm. The Western blot analysis was performed 3 times. Each of the asexual and sexual stages immunofluorescence microscopy experiments were performed 3 times.

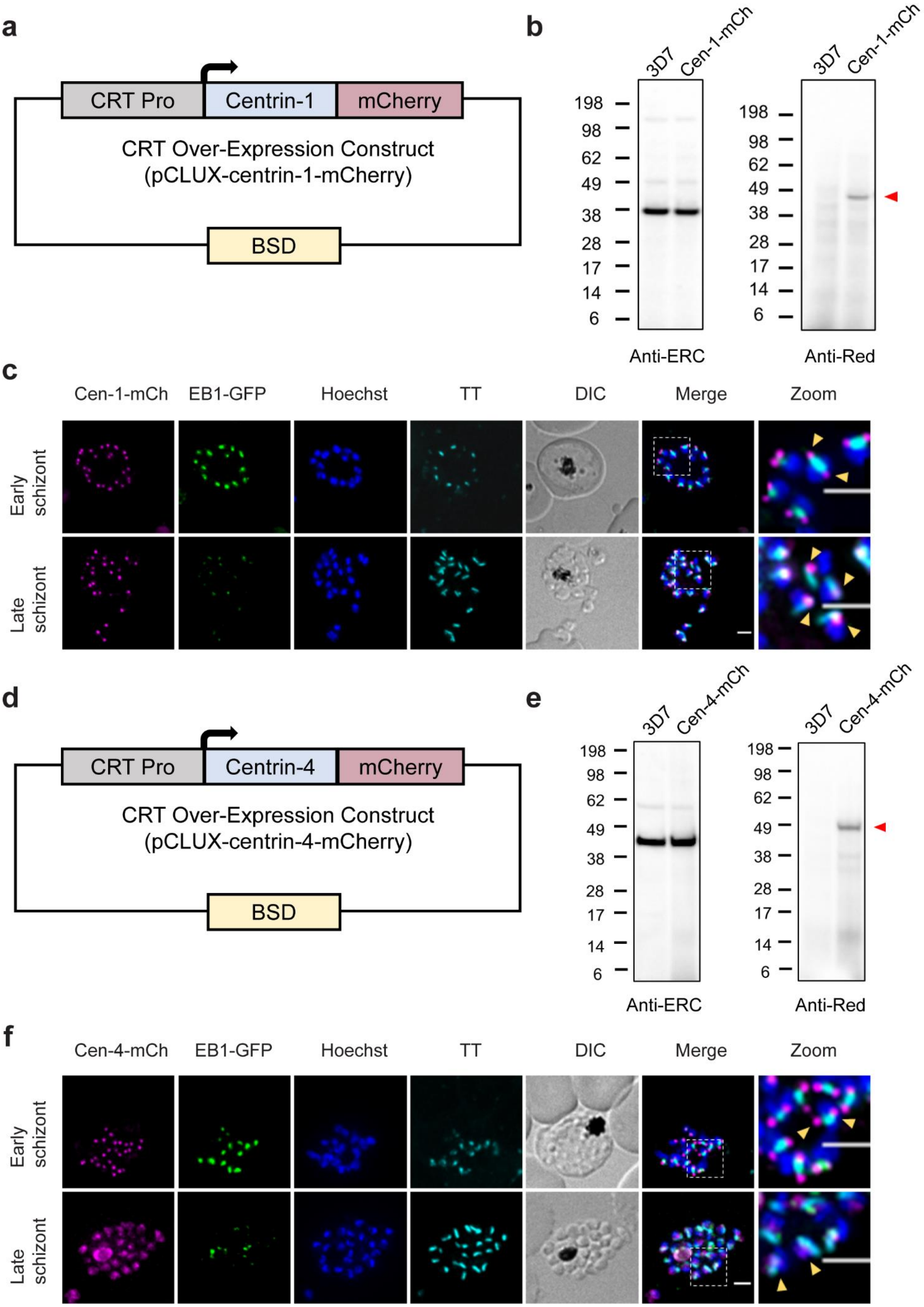

a
CRT Pro
Centrin-1
mCherry
CRT Over-Expression Construct
(pCLUX-centrin-1-mCherry)
BSD
b
3D7
Cen-1-mCh
198
98
62
49
38
28
17
14
6
Anti-ERC
Anti-Red
c
Cen-1-mCh
EB1-GFP
Hoechst
TT
DIC
Merge
Zoom
Early schizont
Late schizont
d
CRT Pro
Centrin-4
mCherry
CRT Over-Expression Construct
(pCLUX-centrin-4-mCherry)
BSD
e
3D7
Cen-4-mCh
Anti-ERC
Anti-Red
f
Cen-4-mCh
EB1-GFP
Hoechst
TT
DIC
Merge
Zoom
Early schizont
Late schizont

Supplementary Figure 5. *Pf*centrin-mCherry-labelled centriolar plaque/MTOC structures in asexual parasites. a Illustration of the construct used to generate the *Pf*centrin-1-mCherry transfectants. **b** Western blot of asexual schizont transfectants, showing a band for *Pf*centrin-1-mCherry (Cen-1-mCh) at the expected molecular mass (red arrow). The parent untransfected cell line (3D7) is used as a control. *Pf*ERC (anti-ERC) is a loading control. **c** Live cell fluorescence imaging of asexual blood stage schizonts from a *Pf*EB1-GFP/*Pf*centrin-1-mCherry co-transfectant line. The data illustrate the relationship between *Pf*centrin-1-mCherry (Cen-1-mCh, magenta), *Pf*EB1-GFP (EB1-GFP, green), Hoechst (blue) and Tubulin Tracker (TT, cyan). Right hand side. Zoom showing short rods of *Pf*EB1-GFP/Tubulin Tracker-labelled spindles, capped by the *Pf*centrin-1-mCherry-labelled centriolar plaque/MTOC (yellow arrows) in dividing nuclei. **d** Illustration of the construct used to generate the *Pf*centrin-4-mCherry transfectants. **e** Western blot of transfectant asexual schizonts expressing *Pf*centrin-4-mCherry (Cen-4-mCh) at the expected molecular mass (red arrow). The parent untransfected cell line (3D7) is used as a control, with *Pf*ERC as a loading control. **f** Live cell fluorescence imaging of asexual blood stage schizonts from a *Pf*EB1-GFP/*Pf*centrin-4-mCherry co-transfectant line, illustrating the relationship of *Pf*centrin-4-mCherry (Cen-4-mCh, magenta), *Pf*EB1-GFP (green), Hoechst (blue) and Tubulin Tracker (TT, cyan). Right hand side. Zoom showing short rods of *Pf*EB1-GFP/Tubulin Tracker-labelled spindles, capped by *Pf*centrin-4-mCherry-labelled centriolar plaque (yellow arrows) in dividing nuclei. Differential interference contrast (DIC) images are shown. Scale bars: 2 µm. The Western blot analysis was performed 3 times for each of the cell lines. The asexual stage live cell imaging experiments were repeated 3 times for each cell line.

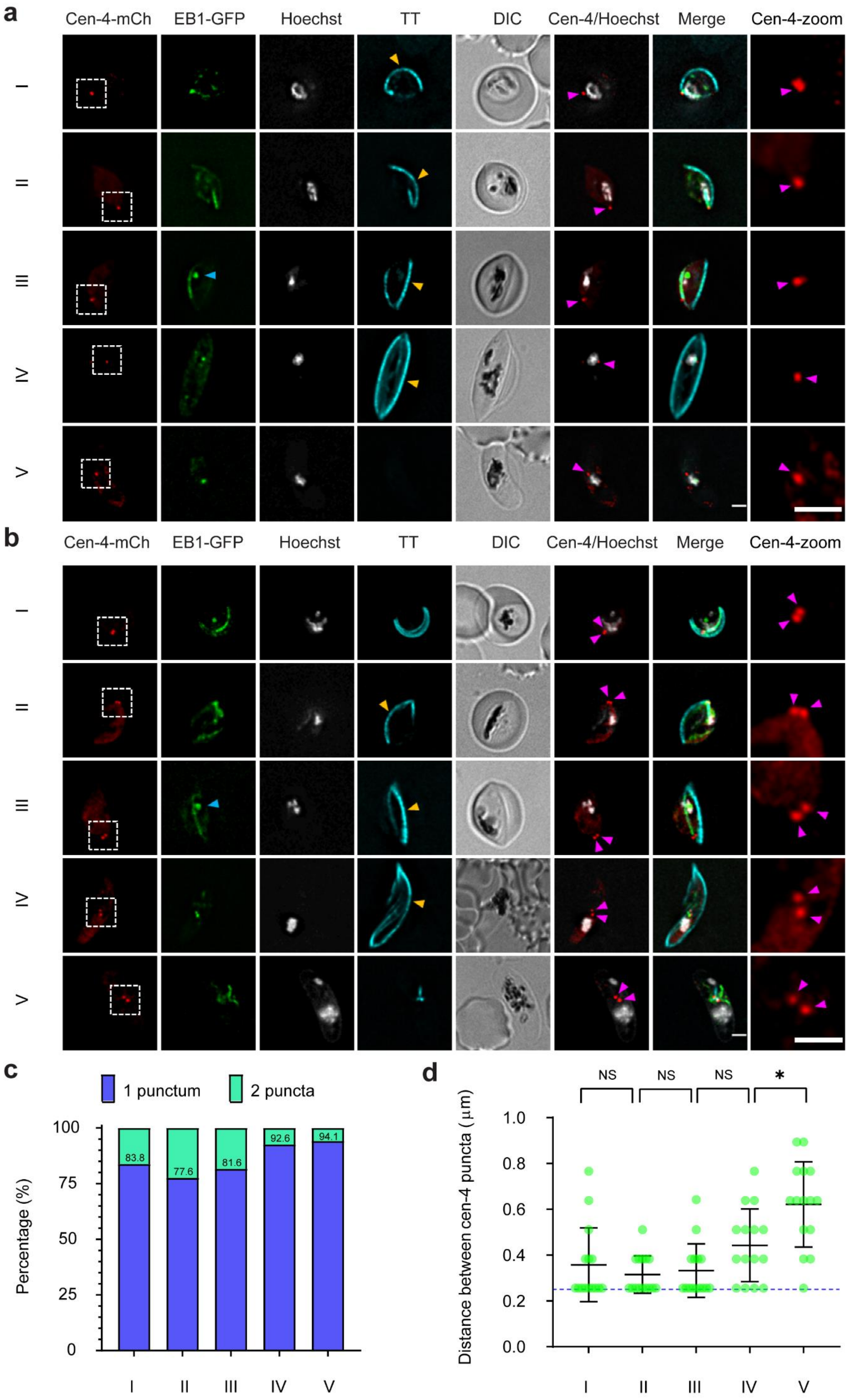

a
Cen-4-mCh
EB1-GFP
Hoechst
TT
DIC
Cen-4/Hoechst
Merge
Cen-4-zoom
I
II
III
IV
V
b
Cen-4-mCh
EB1-GFP
Hoechst
TT
DIC
Cen-4/Hoechst
Merge
Cen-4-zoom
I
II
III
IV
V
c
1 punctum
2 puncta
Percentage (%)
100
75
50
25
0
83.8
77.6
81.6
92.6
94.1
I
II
III
IV
V
d
NS
NS
NS
*
Distance between cen-4 puncta (μm)
1.0
0.8
0.6
0.4
0.2
0.0
I
II
III
IV
V

Supplementary Figure 6. Reorganisation of nuclear microtubule marker, *Pf*EB1-GFP, relative to gametocyte centriolar plaque/MTOC marker, *Pf*centrin-4, and evidence for two *Pf*centrin-4 puncta in a sub-set of gametocytes. a and **b** Live cell fluorescence imaging of stage I-V gametocytes. *Pf*centrin-4-mCherry (Cen-4-mCh, red, magenta arrowheads) marks the punctate centriolar plaque/MTOC. *Pf*EB1-GFP (EB1-GFP, green) decorates the nuclear microtubules, which distinguishes this population from the sub-pellicular microtubules, both labelled with Tubulin Tracker (TT, cyan). The chromatin was labelled with Hoechst (grayscale). The subpellicular microtubules (yellow arrowheads) appear to emanate from the centriolar plaque/MTOC in early stage gametocytes (I/II). As the gametocyte develops, the connection between the nuclear and sub-pellicular microtubule populations is lost (stage III). In stage IV, the sub-pellicular microtubules form a cage around the gametocyte while the nuclear microtubules collapse. The centriolar plaque is present as two puncta in some cells (**b**). In stage V, the sub-pellicular microtubules disassemble. Differential interference contrast (DIC) images are shown. Scale bars: 2 μm. Panel a, b represents additional examples of the data presented in Fig. 3a. **c** Quantitative analysis of the number of centrin puncta at different stages of development ($n \geq 37$ cells in each stage). The number on each bar represents the percentage of cells with one punctum. The experiments were performed three times using different gametocyte batches. **d** The distance between the two *Pf*centrin-4-mCherry puncta was quantified at different stages of development ($n = 15$ cells for each stage). The differences between the distances were analysed using an unpaired one-way ANOVA Turkey's test. I vs. II: NS = 0.9300; II vs. III: NS = 0.9976; III vs. IV: NS = 0.2429; IV vs. V: *p = 0.0109. Source data for Supplementary Fig. 6 c, d is provided in the Source Data file.

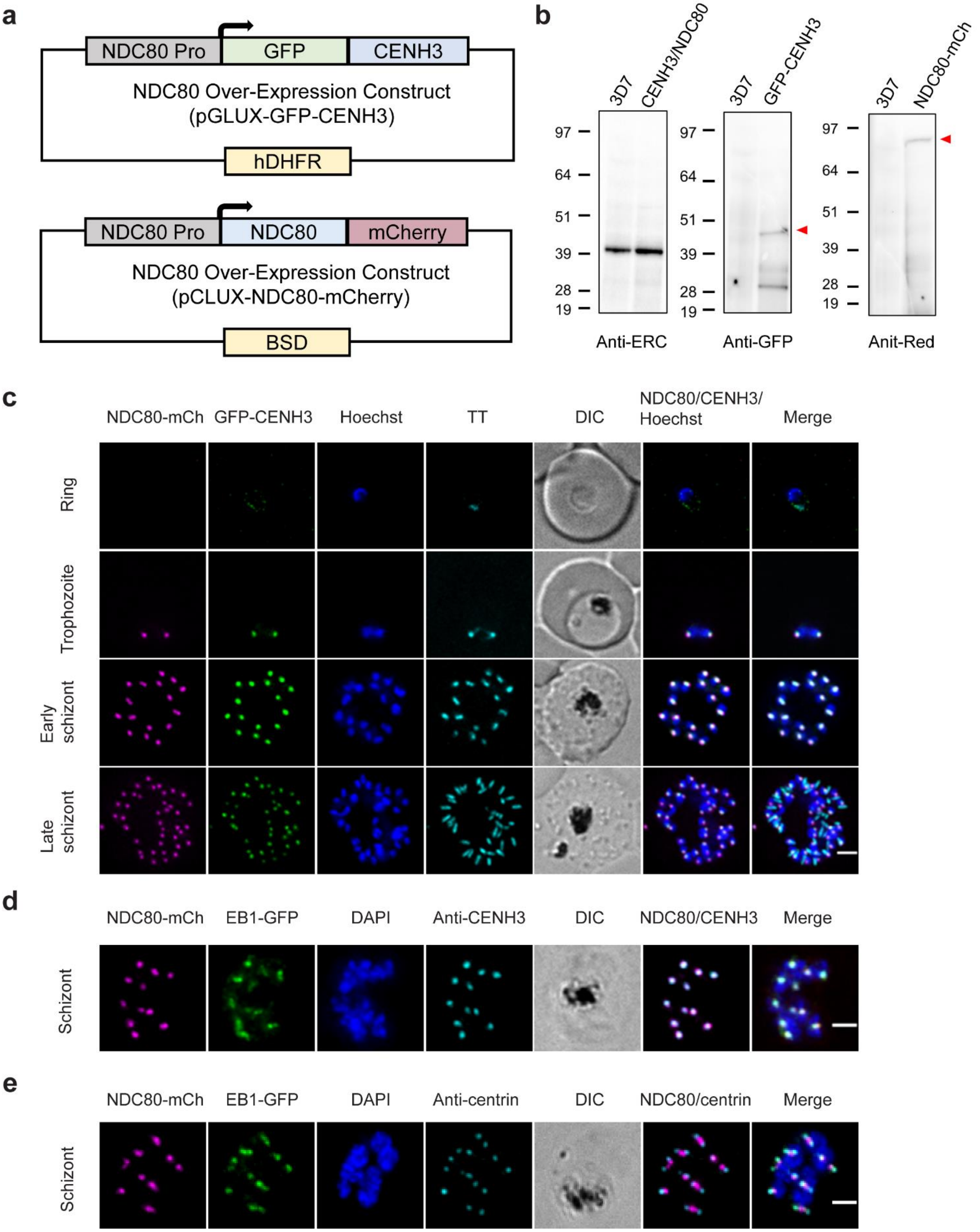

Supplementary Figure 7. GFP-*Pf*CENH3/*Pf*NDC80-mCherry co-transfectants as markers of centromeres and kinetochores in *P. falciparum* asexual stages. a Constructs for over-expression of GFP-*Pf*CENH3 and *Pf*NDC80-mCherry. **b** Western blots showing GFP-*Pf*CENH3 and *Pf*NDC80-mCherry (NDC80-mCh) expressed at the expected molecular masses (red arrows). **c** Live cell images

of GFP-*Pf*CENH3/*Pf*NDC80-mCherry co-transfectants in ring, trophozoite and schizont stages. The centromere (GFP-*Pf*CENH3, GFP-CENH3, green) and kinetochore (*Pf*NDC80-mCherry, NDC80-mCh, magenta) can be observed from trophozoite to mature schizont. The spindle/subpellicular microtubules were labelled with Tubulin Tracker (TT, cyan) and the chromatin was marked by Hoechst (blue). Scale bar: 2 μm. **d** Immunofluorescence of *Pf*NDC80-mCherry (NDC80-mCh, magenta)/*Pf*EB1-GFP (EB1-GFP, green) co-transfectant schizont co-labelled with an antibody recognising *Pf*CENH3 (anti-CENH3, cyan). **e** Immunofluorescence image of *Pf*NDC80-mCherry/*Pf*EB1-GFP co-transfectant schizont co-labelled with an antibody recognising centrin (cyan). The chromatin was marked with DAPI (blue). Differential interference contrast (DIC) images are shown. Scale bar: 2 μm. The Western blot analysis was repeated 3 times. The live cell and immunofluorescence experiments were performed 3 times.

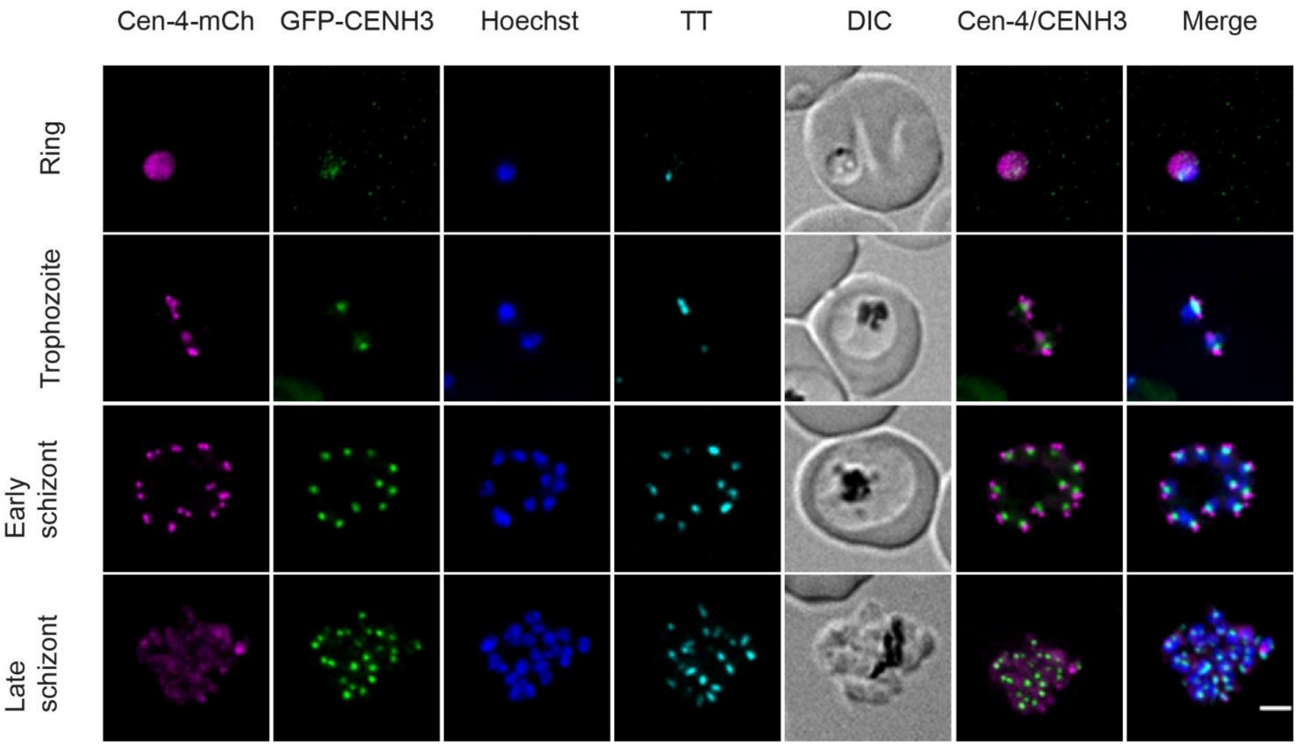

Supplementary Figure 8. The centromere marker, GFP-*Pf*CENH3, remains adjacent to the centriolar plaque/MTOC marker, *Pf*centrin-4-mCherry, in dividing *P. falciparum* asexual stages. Live cell images of GFP-*Pf*CENH3/*Pf*centrin-4-mCherry co-transfectants in ring, trophozoite and schizont stages. The centriolar plaque/MTOC (*Pf*centrin-4-mCherry, Cen-4-mCh, magenta) is evident from trophozoite stage, while the centromere (GFP-*Pf*CENH3, CENH3, green) is evident from ring stage to mature schizont. The spindle/subpellicular microtubules were labelled with Tubulin Tracker (TT, cyan) and the chromatin was marked by Hoechst (blue). Differential interference contrast (DIC) images are shown. Scale bar: 2 μm. The live cell imaging experiments were repeated 3 times.

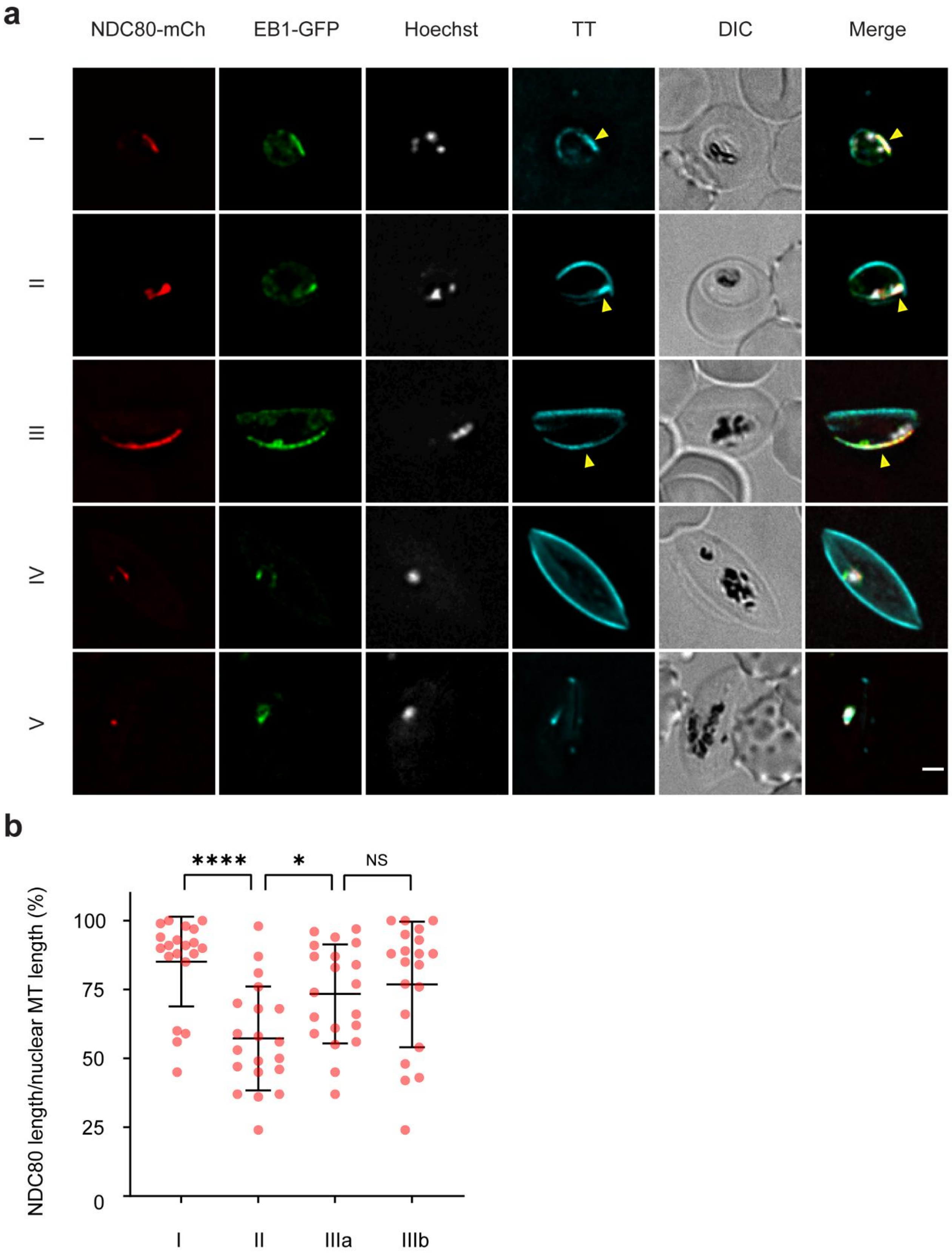

Supplementary Figure 9. Overlap of the kinetochore and nuclear microtubules markers during maturation of *P. falciparum* gametocytes. a Additional live cell images of *Pf*NDC80-mCherry /*Pf*EB1-GFP co-transfectants, covering stage I to stage V gametocytes. The kinetochore (marked by

*Pf*NDC80-mCherry, NDC80-mCh, red) is enriched along the nuclear microtubules (*Pf*EB1-GFP, EB1-GFP, green), also overlapping with chromatin (labelled with Hoechst, grayscale). As the nuclear microtubules disassemble, in stage IV and stage V gametocytes, the kinetochore is evident as a bright point near the area of the chromatin and *Pf*EB1-GFP. Differential interference contrast (DIC) images are shown. Scale bar: 2 µm. **b** Quantitative analysis of the contour lengths of the *Pf*NDC80-mCherry and nuclear microtubule (MT) Tubulin Tracker (TT, cyan, yellow arrows) profiles showing that *Pf*NDC80-mCherry occupies 74% of the nuclear microtubule length (experimental data are presented as mean ± SD; n = 20 cells per stage). Differences in the ratio of *Pf*NDC80/nuclear microtubule were determined by unpaired one-way ANOVA Tukey's test. I vs. II: ****p < 0.0001; II vs. IIIa: *p = 0.0393; IIIa vs. IIIb: NS = 0.8246. Source data for Supplementary Fig. 9b is provided in the Source Data file.

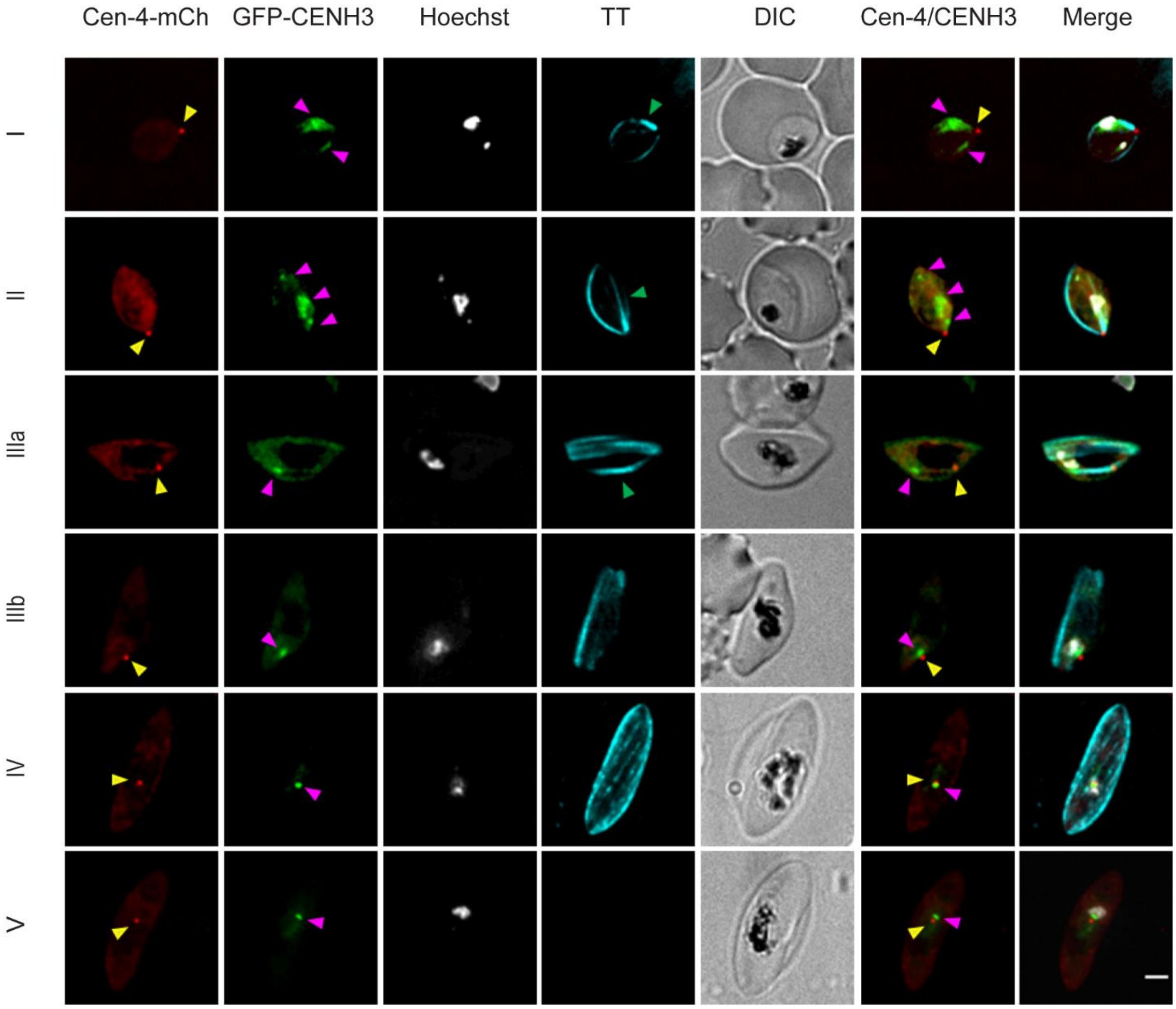

Supplementary Figure 10. Relative locations of the centromere and centriolar plaque/MTOC and nuclear microtubule markers during maturation of *P. falciparum* gametocytes. Additional representative live cell images (related to Fig. 4e) showing GFP-*Pf*CENH3/*Pf*centrin-4-mCherry co-transfectants at different stages of gametocyte development. The distance between the centriolar plaque/MTOC (*Pf*centrin-4-mCherry, Cen-4-mCh, red, yellow arrows) and the centromere (GFP-*Pf*CENH3, GFP-CENH3, green, magenta arrows) increases as elongated nuclear microtubules (Tubulin Tracker (TT), cyan, green arrows) form in stage II gametocytes. Following disassembly of nuclear microtubules in the late stage gametocytes, the centromere moves back towards the centriolar plaque/MTOC. The chromatin was labelled by Hoechst (grayscale). Differential interference contrast (DIC) images are shown. Scale bar: 2 µm. The live cell imaging experiments were performed 3 times.

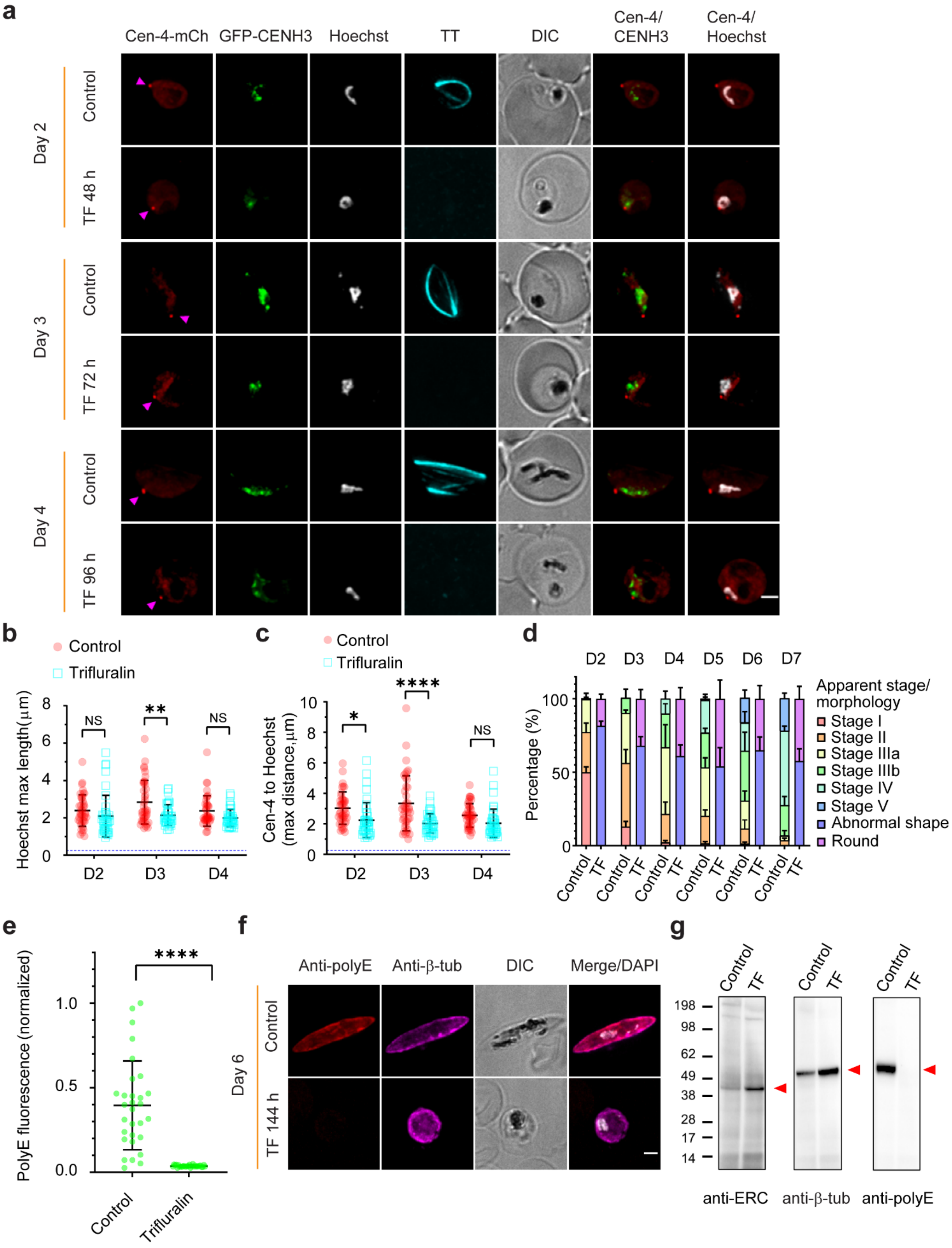

Supplementary Figure 11. Trifluralin treatment of ring stage gametocytes disrupts gametocyte morphology and chromatin spatial organization, as revealed by centriolar plaque and

centromere markers. a Ring stage gametocyte expressing *Pf*centrin-4-mCherry (Cen-4-mCh, MTOC; red) and GFP-*Pf*CENH3 (GFP-CENH3, centromere; green) reporters were treated with or without trifluralin (TF, 5 μM). Images of samples from 48 h (Day 2) to 96 h (Day 4) reveal that both nuclear and subpellicular microtubules are ablated (Tubulin Tracker, TT, cyan), causing the gametocytes to adopt a round shape. The chromatin was marked by Hoechst (grayscale). Differential interference contrast (DIC) images are shown. Scale bar: 2 μm. Images represent additional examples to those shown in Fig. 5a. **b** Maximum length of the Hoechst feature and **c** distance from *Pf*centrin-4-mCherry (cen-4) punctum to furthest Hoechst feature (mean ± SD is shown, n = 40 cells/Day). Statistical differences in the Hoechst maximum length and the maximum distance between *Pf*centrin-4 punctum and furthest Hoechst feature were determined by two-way ANOVA Tukey's test. In Supplementary Fig. 11b, D2: NS = 0.6419; D3: **p = 0.0393; D4: NS = 0.8246. In Supplementary Fig. 11c, D2: *p = 0.0229; D3: ****p < 0.0001; D4: NS = 0.3016. **d** Quantitative analysis of apparent morphological stage (data represent the mean percentage of each stage ± SD, n = 3 experiments) gametocytes in control and trifluralin-treated samples. **e** Quantitation of polyglutamate (polyE) immunofluorescence on Day 6 of development (mean ± SD is shown, n = 30 cells for each group). Differences in polyE fluorescence were assessed using a two-tailed unpaired t test, ****p < 0.0001. **f** Immunofluorescence of Day 6 gametocytes showing labeling for polyE (red) and β tubulin (anti-β tub, magenta). The chromatin was marked by DAPI (grayscale). Scale bar: 2 μm. **g** Control and trifluralin-treated gametocytes were harvested on Day 7 and subjected to Western transfer. The blot was probed with anti-polyE, anti-β tubulin (anti-β tub) and anti-*Pf*ERC (loading control), with stripping between antibody applications. Source data for Supplementary Fig. 11b-e is provided in the Source Data file. The immunofluorescence and western blot experiments were performed 2 times.

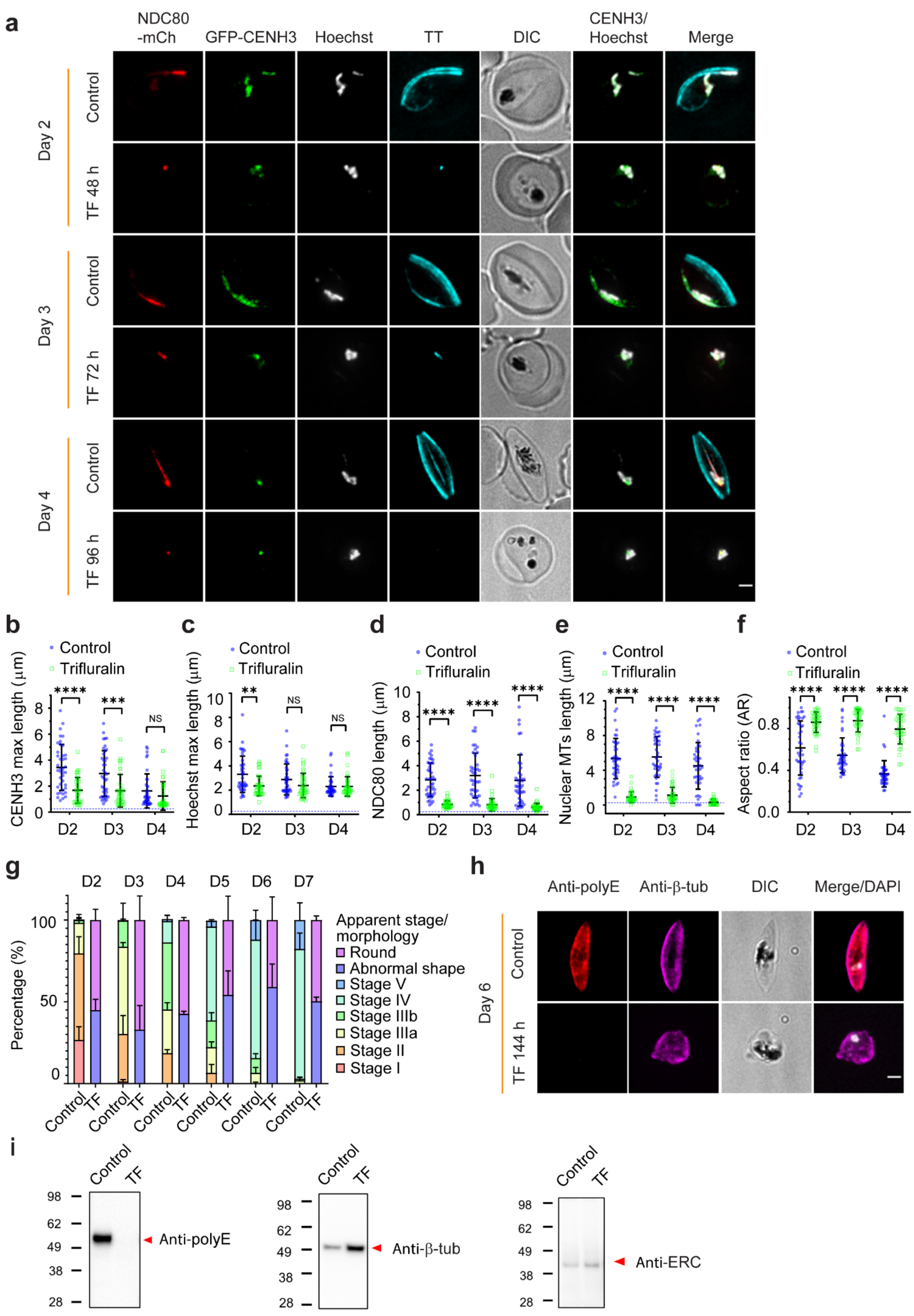

a
NDC80 -mCh
GFP-CENH3
Hoechst
TT
DIC
CENH3/ Hoechst
Merge
Day 2
Control
TF 48 h
Day 3
Control
TF 72 h
Day 4
Control
TF 96 h
b
Control
Trifluralin
CENH3 max length (µm)
****
***
NS
D2
D3
D4
c
Hoechst max length (µm)
**
NS
NS
d
NDC80 length (µm)
****
e
Nuclear MTs length (µm)
f
Aspect ratio (AR)
g
Percentage (%)
D2
D3
D4
D5
D6
D7
Control
TF
Apparent stage/ morphology
Round
Abnormal shape
Stage V
Stage IV
Stage IIIb
Stage IIIa
Stage II
Stage I
h
Anti-polyE
Anti-β-tub
DIC
Merge/DAPI
Day 6
Control
TF 144 h
i
Control
TF
Anti-polyE
Anti-β-tub
Anti-ERC
98
62
49
38
28

Supplementary Figure 12. Trifluralin treatment of ring stage gametocytes disrupts cellular remodeling, as revealed by kinetochore and centromere markers. A Ring stage gametocyte transfectants expressing *Pf*NDC80-mCherry (NDC80-mCh, kinetochore marker; red) and GFP-*Pf*CENH3 (GFP-CENH3, centromere marker; green) were treated with or without trifluralin (TF, 5 μM). Images of 48 h (Day 2), 72 h (Day 3) and 96 h (Day 4)- treated cells reveal the disassembly of nuclear and subpellicular microtubules, causing the gametocytes to adopt a round or abnormal shape and resulting in decreased dispersion of the kinetochore and centromere markers. The chromatin was marked by Hoechst (grayscale). Differential interference contrast (DIC) images are shown. Scale bar: 2 μm. **B, c** Maximum length of the *Pf*CENH3 (CENH3) (**b**) and Hoechst (**c**) features (mean ± SD is shown, n = 40 cells/Day). Statistical differences of the maximum length of *Pf*CENH3 and Hoechst features were evaluated by two-way ANOVA Tukey's test. In Supplementary Fig. 12 b, D2: ****p < 0.0001; ***p = 0.0004, NS = 0.8093. In Supplementary Fig. 12c, D2: **p = 0.0011; D3: NS = 0.2995; D4: NS > 0.9999. **d, e** Assessment of the contour length of the *Pf*NDC80-mCherry (NDC80) profile (d) and nuclear microtubules (nuclear MTs, Tubulin Tracker, TT, green arrows) (e) (mean ± SD is shown, n = 40 cells/Day). Differences in the length of *Pf*NDC80 and nuclear microtubule features were assessed by two-way ANOVA Tukey's test, ****p < 0.0001. **f** Quantitative analysis of aspect ratio (mean ± SD is shown, n = 40 cells/Day). **g** Morphological stage distribution (mean ± SD, n = 3 experiments). Differences in the aspect ratio were determined by two-way ANOVA Tukey's test, ****p < 0.0001. **h** Anti-polyE (red) signals in control and trifluralin-treated gametocytes (Day 6). Polymerized or depolymerized microtubules were labelled by anti-β tubulin (anti-β tub, magenta). The chromatin was marked by DAPI (grayscale). **i** Western blots of Day 7 control and trifluralin-treated gametocytes probed with anti-β tubulin (anti-β tub), anti-polyE and anti-*Pf*ERC (anti-ERC, loading control). Source data for Supplementary Fig. 12b-g is provided in the Source Data file. The immunofluorescence and western blot experiments were performed 2 times.

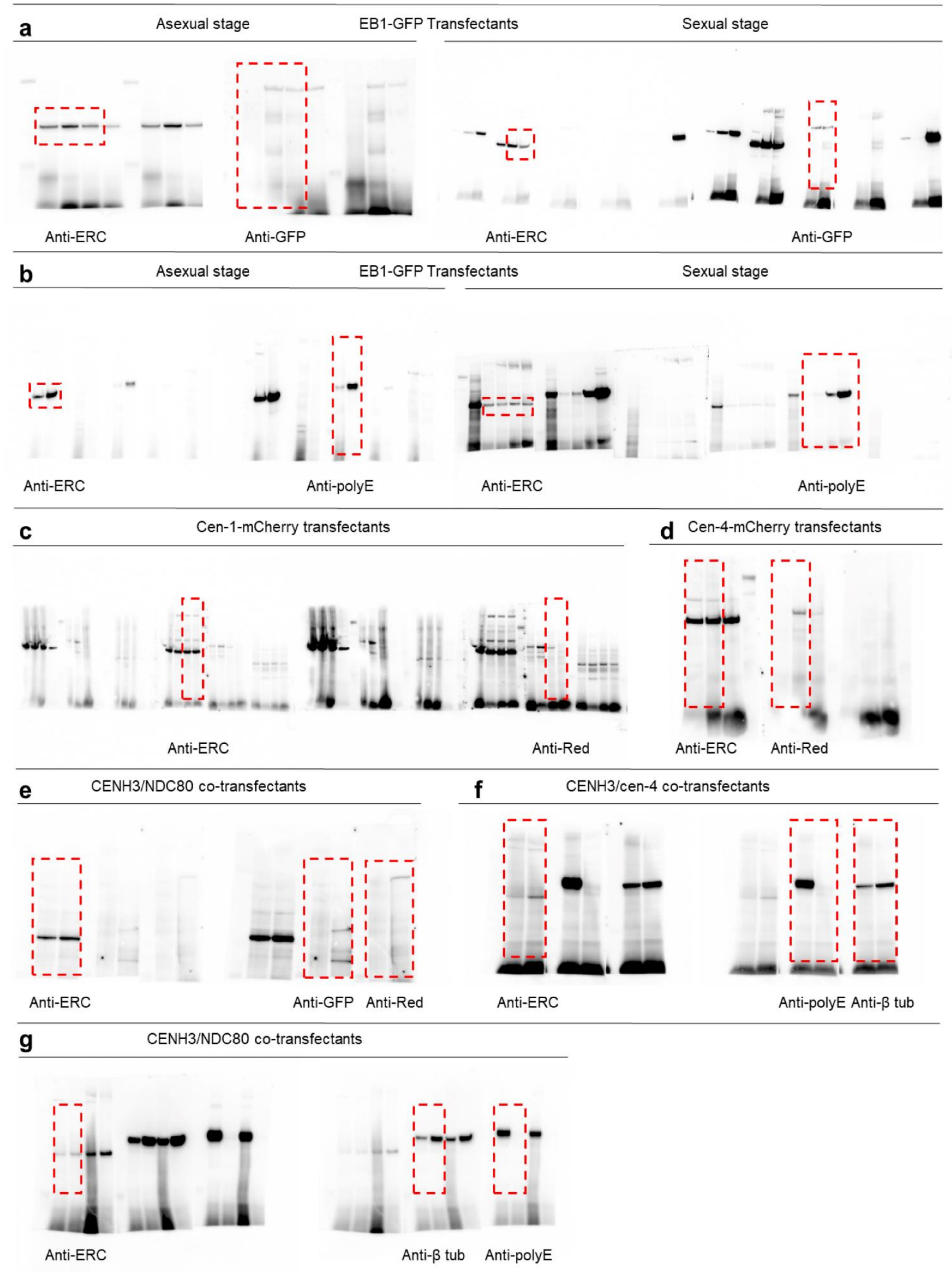

Supplementary Figure 13. Uncropped Western blot images. a Blots relating to Supplementary Fig. 3b, showing *Pf*EB1-GFP (EB1-GFP) transfectants, asexuals (left) and gametocytes (right), probed with

anti-*Pf*ERC (anti-ERC) and anti-GFP. **b** Blots relating to Supplementary Fig. 4a and 4c showing *Pf*EB1-GFP transfectants, asexuals (left) and gametocytes (right), probed with anti-ERC and anti-polyE. **c** Blots relating to Supplementary Fig. 5b showing *Pf*centrin-1-mCherry (cen-1-mCherry) asexual transfectants, probed with anti-ERC and anti-Red. **d** Blots relating to Supplementary Fig. 5e showing *Pf*centrin-4-mCherry (cen-4-mCherry) asexual transfectants, probed with anti-ERC and anti-Red. **e** Blots relating to Supplementary Fig. 7b, showing *Pf*GFP-CENH3/*Pf*NDC80-mCherry (CENH3/NDC80) co-transfectants, probed with anti-ERC, anti-GFP and anti-Red. **f** Blots relating to Supplementary Fig. 11g, showing *Pf*GFP-CENH3/*Pf*centrin-4-mCherry (CENH3/cen-4) co-transfectants, probed with anti-ERC, anti-polyE and anti-β-tubulin. **g** Blots relating to Supplementary Fig. 12i, showing *Pf*GFP-CENH3/*Pf*NDC80-mCherry (CENH3/NDC80) co-transfectants, probed with anti-ERC, anti-β-tubulin and anti-polyE.

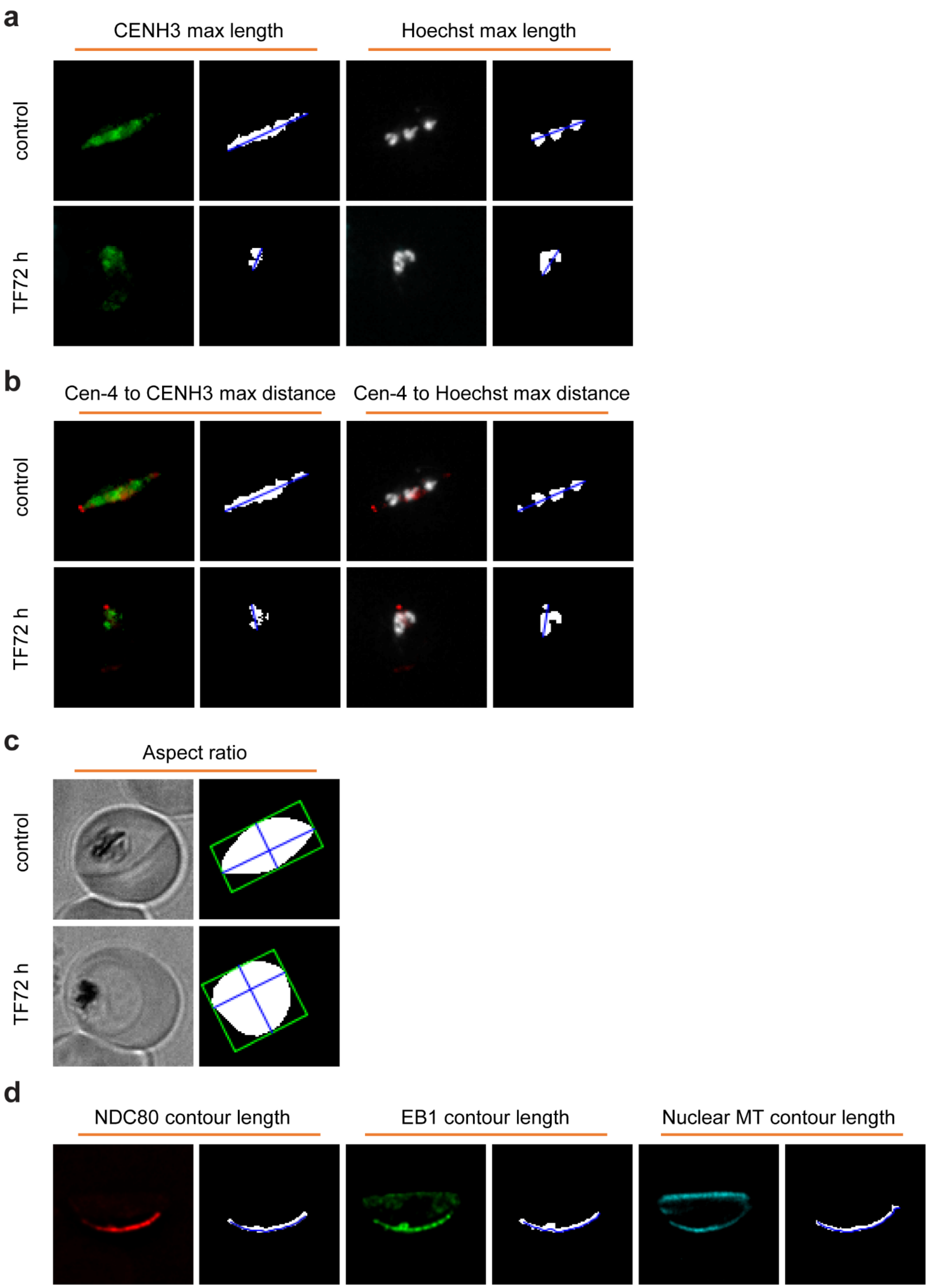
a
CENH3 max length
Hoechst max length
control
TF72 h
b
Cen-4 to CENH3 max distance
Cen-4 to Hoechst max distance
control
TF72 h
c
Aspect ratio
control
TF72 h
d
NDC80 contour length
EB1 contour length
Nuclear MT contour length

Supplementary Figure 14. Schematic illustration of the method to measure maximum distance, contour length and aspect ratio. Panels from Fig. 5a (Day 3) and Supplementary Fig. 9a (Stage III) overlayed with lines illustrating the method for estimating maximum distance, i.e., distance between the furthermost features; or contour length, i.e., measurement following the shape of the fluorescent structure, where possible; and aspect ratio, i.e., maximum cell length divided by minimum cell width. **a** Maximum length measurement illustrated using data for GFP-*Pf*CENH3 (CENH3, centromere; green) and Hoechst (chromatin; grayscale). The original image data and the processed data are presented. The Maximum Feret Diameter was determined using via the MorphoLibJ plugin in FIJI. **b** Maximum distance measurements. The distance from the *Pf*centrin-4-mCherry (Cen-4, MTOC; red) punctum to the furthermost point of the GFP-*Pf*CENH3 (CENH3, green) or Hoechst (grayscale) fluorescence signal was measured. The original data and the processed data are presented. For the analysis the two targeted channels were combined; and the Maximum Feret Diameters was measured using the MorphoLibJ plugin. **c** Aspect ratio measurements. The Differential interference contrast channel was used to generate an oriented Bounding Box (using the MorphoLibJ plugin). The width and length of the box was then measured. **d** Contour length measurements illustrated for *Pf*NDC80-mCherry (NDC80, kinetochore; red), *Pf*EB1-GFP (EB1, nuclear microtubule marker; green) and nuclear microtubules (nuclear MT, Tubulin Tracker; cyan) in *Pf*NDC80-mCherry/*Pf*EB1-GFP gametocytes. The original and processed data are presented. The contour length was measured with Geodesic Diameter (using the MorphoLibJ plugin).

References

1. Birnbaum J*, et al.* A genetic system to study *Plasmodium falciparum* protein function. *Nat Methods* **14**, 450-456 (2017).
