# Supplementary material for: Repurposing the mitotic machinery to drive cellular elongation and chromatin reorganisation in Plasmodium falciparum gametocytes
Source: Nat Commun. 2022 Aug 27;13:5054. doi: 10.1038/s41467-022-32579-4 (PMC9419145; doi:10.1038/s41467-022-32579-4)
Supplement: Supplementary file 3 — Description of Additional Supplementary Information [file 41467_2022_32579_MOESM3_ESM.pdf]

Description of Additional Supplementary Information File

Title: Supplementary Movies 1-3:

Description: Serialsection TEM of stage I to III gametocytes. Videos showing translations through serial sections and rendered models of stage I - III gametocytes (Movies 1-3). The nuclear microtubule bundles emanate from an amorphous centriolar plaque/MTOC. Chromatin material is attached to the microtubule bundle. Legend of rendered colours: Green, parasite plasma membrane; blue, outer nuclear membrane; red, centriolar plaque/MTOC; yellow, microtubules; gold, chromatin. Scale bars = 1 µm.

Title: Supplementary Movie 4 and 5:

Description: Electron Tomography of stage II (Movie 4) and III (Movie 5) gametocytes. Translations through virtual sections and rendered models of the region where a bundle of intranuclear microtubules emanates from the centriolar plaque/MTOC. The centriolar plaque/MTOC is embedded within the nuclear membrane with density extending into the cytoplasm. The MTOC is close to a bulged region of the nuclear membrane that appears to be connected to an endoplasmic reticulum extension and to be the site of initiation of the IMC. Legend of rendered colours: Green, parasite plasma membrane; blue, outer nuclear membrane; cyan, inner nuclear membrane; magenta, ER; grey, IMC; red, centriolar plaque/MTOC; yellow, microtubules.
